# Duchenne Muscular Dystrophy in the Republic of North Ossetia–Alania: Epidemiological Study, Diagnostic Issues, and Treatment Prospects

**DOI:** 10.3390/genes16121458

**Published:** 2025-12-06

**Authors:** Rena Zinchenko, Inna Tebieva, Aysylu Murtazina, Sofya Ionova, Alisa Zhmurova-Kriventsova, Olga Shchagina, Elena Zinina, Yulia Gabisova, Alana Khokhova, Marina Tokazova, Murat Ikaev, Oleg Remizov, Sofia Popovich, Ludmila Kuzenkova, Andrey Marakhonov, Sergey Kutsev

**Affiliations:** 1 Research Centre for Medical Genetics, Moskvorechye St., Moscow 115522, Russia; 2 Federal State Budgetary Educational Institution of Higher Professional Education “North-Ossetian State Medical Academy” of the Ministry of Health of the Russian Federation, Vladikavkaz 362019, Russia; 3 Republican Children’s Clinical Hospital of the Republic of North Ossetia-Alania, Republic of North Ossetia-Alania, St. Pushkinskaya 40, Vladikavkaz 362007, Russia; 4 Federal State Autonomous Institution “National Medical Research Center for Children’s Health” of the Ministry of Health of the Russian Federation, Lomonosovsky Prospect, Moscow 119991, Russia

**Keywords:** Duchenne muscular dystrophy, targeted therapy, gene replacement therapy

## Abstract

Background/Objectives: The article presents data on Duchenne muscular dystrophy (DMD) in the Republic of North Ossetia-Alania (RNOA), describing the population characteristics of the disease among children in RNOA (2006–2023). Methods: The number of newborns was 172,115, with 86,057 boys from 2006 to 2023. During the reporting period, 19 families (23 patients, including 22 boys) were identified. The molecular and genetic characteristics of the patients were analyzed throughout the entire observation period, which began in 1998. Results: The prevalence of the disease was 1:3912 among newborn boys (95%CI: 1:2584–1:6242), which is slightly higher than in other regions of the Russian Federation (RF) and most countries around the world. The spectrum of *DMD* genetic variants in RNOA aligns with worldwide patterns but reveals differences in frequencies compared to RF data. The frequency of exon deletions in the *DMD* gene range from 65.0 to 70.0% (60% in RNOA vs. <50% in RF) worldwide, for duplications—9.0–11.0% globally (16% in RNOA), and for nonsense variants—9.7–26.5% worldwide (20% in RNOA). Twelve patients (41.0%) in RNOA qualified for therapy, and Translarna was prescribed in most cases. In the cohort of children, one girl was identified with classic DMD, confirmed by genetic studies. Different limitations of the study were hindered by the small cohort size, patients’ remote residences, and poor therapy compliance of our patients. Conclusions: The heterogeneity of mutation spectrum across different populations underscores the influence of ethnic background. Consequently, this study highlights the importance of population-specific studies for improving DMD care.

## 1. Introduction

Duchenne/Becker muscular dystrophy (DMD/BMD) is the most common form of muscular dystrophy worldwide, with the frequency ranging from 1:3500 to 1:5000 in newborn boys (OMIM #310200, #300376). In rare cases, female carriers can also manifest symptoms of the disease [1,2,3,4,5]. Based on these prevalence data, the estimated number of DMD/BMD patients in the Russian Federation is about 4000 [6]. The French scientist G.B.A. Duchenne first described the classic clinical presentation of DMD in 1861, who referred to the disease as “paralytic muscular pseudohypertrophy” [7]. A clinical form of muscular dystrophy with later onset and milder course (BMD) was first described by another scientist, P.E. Becker, in 1964 and occurs with a frequency of 1 in 20,000 boys worldwide [8,9].

DMD/BMD is an X-linked recessive disorder caused by pathogenic variants in the DMD gene (OMIM *300377), which encodes dystrophin, which is localized on the cytoplasmatic side of sarcolemma. This protein is part of a large dystrophin-glycoprotein complex that links cytoskeletal (actin) and proteins of extracellular matrix. Dystrophin deficiency leads to membrane instability, muscle degeneration, and necrosis of muscle fibers. More than 4700 genetic variants have been described: large deletions accounting for ~55–65% of cases, duplications for ~10%, and the remaining cases are caused by single-nucleotide variants, of which 10–15% are nonsense variants [9,10,11,12]. In 2022, Salari et al. published a global study on the prevalence of muscular dystrophy across continental subgroups (Asia, Europe, Africa, and America), reporting an average of 3.6 cases per 100,000 people (95% CI (Confidence interval): 2.8–4.5) [13]. Their analysis estimated the prevalence of DMD at 4.8 (95% CI: 3.6–6.3) and for BMD at1.6 (95% CI: 1.1–2.4) per 100,000 people, respectively. Globally, the prevalence of Duchenne muscular dystrophy (DMD) ranged from 0.7% to 16.7% [13].

The algorithm for diagnosing DMD in Russia aligns with global standards and includes measuring the levels of alanine aminotransferase (ALT), aspartate aminotransferase (AST), creatine phosphokinase (CK), lactate dehydrogenase (LDH), and molecular genetic testing. Functional activity is assessed using the six-minute walk test (6MWT), the North Star Ambulatory Assessment (NSAA), and others [3,14].

Overall, Russian clinical guidelines are consistent with international standards. The “gold standard” of therapy for DMD/BMD is the use of systemic corticosteroids (prednisone/deflazacort), which can preserve the ability to walk independently for 3–5 years. The initiation of hormonal therapy depends on the stage of motor function development, the patient’s condition, the presence of complications, and disease progression characteristics. Treatment should be initiated at age 4, without waiting for signs of motor decline. Initiating therapy before age 7 has the greatest impact on prolonging independent ambulation. Starting at age 6, treatment may be expanded to include ACE (angiotensin-converting enzyme) inhibitors, angiotensin II receptor blockers, diuretics, mineralocorticoid receptor antagonists, and beta-blockers. To preserve mobility, management includes vitamin D3, calcium supplements, bisphosphonates, physical therapy, and joint exercises [3].

The advent of modern treatments, including targeted and gene therapies, has significantly increased the importance of early DMD/BMD diagnosis. In the Russian Federation (RF), the “Circle of Kindness” foundation (Foundation for Support of Children with Severe Life-Threatening and Chronic Diseases, including Rare (Orphan) Diseases) manages the provision of medications for patients. The preference for disease-modifying therapy depends on the specific genetic variant. For nonsense variants, Translarna is used for promoting to overcome the stop codon and recover dystrophin production in ambulatory patients over 2 years of age. This medicine has been included in the foundation’s list since April 2021. For patients with deletion of certain exons, the exon-skipping therapies are available: viltolarsen and golodirsen (for exon 53 deletions), eteplirsen (for exon 51 deletions), and casimersen (for exon 45 deletions). The one-time gene therapy delandistrogene moxeparvovec-rokl is indicated for patients under 8 years of age with all genetic variants except deletions of exons 8 and 9 [15,16,17,18,19].

The aim of this study is the investigation of molecular and population-specific features of DMD/BMD in the Republic of North Ossetia-Alania (RNOA), one of the regions of the RF, and the evaluation of intermediate treatment efficiency.

## 2. Materials and Methods

We conducted a retrospective analysis using medical records from patients diagnosed with DMD/BMD, who were registered at the Medical Genetic Counseling Center of the Republican Children’s Clinical Hospital, Republic of North Ossetia-Alania (RCCH RNOA). The diagnosis was established using clinical and paraclinical data, along with patient history. The laboratory assessment included measurements of liver transaminases (ALT and AST) and CPK levels. Patients with symptoms of DMD/BMD or asymptomatic individuals with elevated CPK levels (>2000 U/L) underwent molecular genetic testing. The molecular genetic diagnostic algorithm consisted of initial screening for large deletions/duplications in the DMD gene using multiplex ligation-dependent probe amplification (MLPA). The presence of large deletions/duplications in the DMD gene served as definitive diagnostic confirmation. For MLPA-negative cases, the second stage was targeted sequencing of the DMD gene as a part of a limb-girdle muscular dystrophy NGS panel or “neuromuscular disease gene panel” testing [3]. The identified variants were confirmed by Sanger sequencing for the proband and their mother. In one case, karyotype analysis was performed. The study did not include MLPA for mothers of patients with Duchenne muscular dystrophy.

For X-chromosome lyonization analysis in a female patient, we assessed methylation patterns of the X-linked HUMARA-(CAG) polymorphic repeat in exon 1 of the AR gene using methylation-sensitive quantitative fluorescent PCR followed by fragment analysis to determine the percentage of cells carrying the inactivated X chromosome [17].

The treatment efficacy was assessed using the 3 or 6 min walk test [9] and the North Star Ambulatory Assessment (NSAA)—the 17-item scale for evaluating functional motor abilities in children with DMD [18]. Drug safety evaluation included monitoring of adverse effects and biochemical parameters according to the medications’ prescribing information.

95% confidence interval (95%CI) was calculated using the Clopper–Pearson method.

## 3. Results

During the period 2006–2023, there were 172,115 live births, among them 86,057 males. The study identified 19 affected families (23 patients, including 22 boys); the disease prevalence was 1 per 3912 male births (95%CI: 1:2584–1:6242) in RNOA, which is higher than reported in other Russian regions and most countries worldwide [3,13,14]. Given the extreme rarity of DMD diagnosis in female children, prevalence calculations were based on male patients only (22 cases from 18 families).

Before 2006, the Medical Genetic Counseling Center in RNOA had documented five patients of Ossetian origin with genetically confirmed DMD with deletions of exons 50–52, 32–43, 45–50, and two patients with deletion of exon 45. Between 2006 and 2023, an additional 23 patients (22 boys and 1 girl) from 19 families with DMD were identified in Republican Children’s Clinical Hospital (RCCH) of RNOA; their clinical and genetic characteristics are presented in Table 1. The cohort included 15 Ossetian families, 3 Russian families, and 1 inter-ethnic family (Ossetian father and Georgian mother). All patients exhibited the DMD phenotype with symptom onset in early childhood.

In the study, structural pathogenic variants affecting one or more exons of the DMD gene were identified in 21 patients (72.41%): deletions in 16 patients (55.17%), duplications in 3 patients (10.34%), and complex rearrangements (duplication of exons 2–9 with duplication of exons 54–56 and deletion exon 7 with duplication of exons 5–6) in 2 patients (6.89%). Nonsense variants were found in 7 patients (27.58%) from 5 families, all previously reported: NM_004006.3(*DMD*):c.2896C > T, p.(Q966*); c.10141C > T (p.R3381*); c.9337C > T (p.R3113*); c.6292 > T (p.R2098); c:7693 C > T (p.Q2565*).

The mean age of symptom onset was 2.7 years, whereas the mean age at diagnosis was 6.6 years. As a result of genetic diagnosis, de novo pathogenic variants were revealed for 12 out of 20 probands, while 8 probands demonstrated positive family history with affected male relatives on the maternal side.

Corticosteroid (CS) therapy was initiated at a dosage of 0.75 mg/kg per day once daily in the morning within the first year after diagnosis in most patients, demonstrating beneficial effects including motor function stabilization and slowed disease progression. However, only 30% of patients received CS treatment during the optimal therapeutic window for motor function preservation (ages 4–7 years). Notably, in 20.8% cases, parents declined CS therapy due to concerns about adverse effects, while in two cases (8.3%), treatment was taken irregularly, primarily because parents discontinued therapy due to a perceived lack of benefit.

Twelve patients (41%) from nine families were recommended for targeted therapy, receiving either Translarna (eight patients) or exon-skipping therapy (five patients). The family № 1/1 (in Table 1) with nonsense variants in probands declined Translarna treatment. Data on targeted treatment for the DMD patient cohort is presented in Table 2. The mean age at initiation of targeted therapy was 7 years. All treated patients underwent an assessment using standard clinical scales at least once; however, only two were evaluated twice—before and during treatment. One patient (№ 16/18 in Table 2) receiving Translarna demonstrated improvement in motor function over 4 years by the 6 min walk test (6MWT) (increase of 82 m). A second patient (№ 19/23 in Table 2) treated with Delandystrogen moxeparvovec-rokl demonstrated improvement in motor function after 4 months on both clinical tests: a 30 m gain on the 6MWT and a 3-point improvement on the North Star Ambulatory Assessment (NSAA). In the middle of 2025, after expanding the age restrictions to 8 years, another patient born in 2016 (№ 17/19 in Table 1) was approved for Delandystrogen moxeparvovec-rokl.

One Russian female patient with DMD was identified in RNOA. The age of debut was 2.5 years, manifested as «toe walking» and frequent falls. The family initially associated these symptoms with recent vaccination. The disease progressed gradually, leading to proximal muscle weakness (difficulty climbing stairs) and arm weakness (trouble carrying even light objects in one hand). Due to significantly elevated CK levels (up to 26,000 U/L) and transaminases, the diagnosis “muscular dystrophy” was suggested. The NGS panel “neuromuscular disease gene panel” was provided but no clinically significant variants were revealed. The next step was MLPA, which revealed a heterozygous deletion of exons 3–11 in the *DMD* gene. Further analysis of X-chromosome inactivation revealed skewed pattern (22%:78%). The combination of factors (classic clinical presentation, the pathogenic *DMD*-variant, unbalanced X-chromosome inactivation, and absence of other molecular genetic causes) allowed us to confirm the diagnosis of DMD in the female patient.

## 4. Discussion

The study included 19 affected families (23 patients, including 22 boys) with DMD in RNOA. The molecular genetic diagnosis was provided for all of them, including one female patient. Targeted therapy, including exon skipping and premature stop-codon therapy (Translarna) were applied to 12 patients. The available data on six patients with DMD/BMD identified before 2006 indicate underdiagnosis of DMD/BMD in the late 1990s and early 2000s, which was probably due to limitations of molecular genetic diagnosis. Two of these patients died at age 20, while four are currently in the late non-ambulatory stage.

We report a case of DMD diagnosis in a female patient. According to the literature data, the diagnosis of DMD in female patients is rare and may be caused not only by unbalanced X-chromosome inactivation (exceeding the 30:70% ratio) but also by Turner syndrome, the combination of DMD with testicular feminization syndrome, or homozygous/hemizygous mutations in both X chromosomes. While limb-girdle muscular dystrophies [13] often mimic DMD phenotypically, DMD should not be excluded from the differential diagnosis in girls with clinical signs of muscular dystrophy and extremely high CK levels.

Analysis of the spectrum of DMD genetic variants in RNOA revealed differences in frequencies compared to Russian Federation (RF) data [9]; however, the frequency of DMD genetic variants in the patient’s cohort from RNOA is aligning with global patterns. The frequency of exon deletions in the DMD gene range is 65.0–70.0% (55% in RNOA vs. <50% in RF) worldwide, for duplications—9.0–11.0% globally (10% in RNOA), and for nonsense variants—9.7–26.5% worldwide (27% in RNOA) [6,20,21].

Based on the literature data, most of the deletions in the DMD gene arise in two hotspots (exons 2–19 and exons 44–53). In 11/15 cases (73%), the presence of deletions, which affect the “hotspot” in the central rod domain of dystrophin (the dystrophin core domain), is registered. In one familial case, two siblings had a deletion of exons 8–17. In two cases, complex rearrangements (dup ex 2–9 with dup ex 54–56 and del ex 7 with dup ex 5–6) were registered. The frequency of nonsense variants exceeds worldwide data (10–15% of DMD/BMD cases) but corresponds to Russian data [9,22,23]. It should be noted that the North Caucasus District, which includes the Republic of North Ossetia-Alania, ranks second to last in terms of the number of patients (36, 5.8%).

The heterogeneity of the mutation spectrum in different populations confirms the influence of ethnic origin, which was further demonstrated in a study by R. Selvatici and colleagues, where an analysis of data from 12 countries revealed significant interethnic differences [24]. In Italy, data were published on deletions in 65% of 1162 patients with DMD/BMD, while 10% had duplications and 25% had small mutations. Nonsense variants (11%) were the most common, followed by small insertions–deletions with frame shift (7%), while 4%, 1%, and 2% included splicing sites, consensus, and rare missense mutations, respectively [25].

At present, it is difficult to determine the exact cause of the differences in mutation spectrum between this study and global data. It should be noted that for a long time, DMD/BMD diagnosis in the RF was limited to detecting the most common rearrangements by MLPA. In 2015, the Research Centre for Medical Genetics implemented small mutation detection using NGS which allowed to determine small mutations presented with high frequency in cohort.

These results demonstrate that in the RNOA, DMD diagnosis is confirmed at a mean age of 6 years following symptom onset at 2 years, which is 1.5 years earlier than the national Russian average diagnostic age of 7.5 years.

Modern treatment methods [26] are available to patients and actively used in the RNOA. According to study data, currently available target therapy is suitable for 34.3% of patients, which includes exon-skipping therapy (51, 53, 45) and premature stop-codon therapy (Translarna). The Treat-NMD registry indicates that approximately 39.0% of patients qualify for therapy, which is consistent with our findings (41% of DMD patients in North Ossetia-Alania). Translarna was prescribed in most cases.

Despite the short follow-up period, one patient treated with Delandistrogene moxeparvovec-rokl demonstrated improvement in both 6 min walk test (6MWT) and North Star Ambulatory Assessment (NSAA) scores at the 4-month assessment. In another case where reevaluation was performed, Translarna therapy resulted in a stable, unchanged neurological status of the child after 4 years of treatment.

### Limitations

This study has several limitations. The primary constraint is the small sample size, which limits statistical power and prevents meaningful population-based or comparative analyses. The discussion of clinical findings is further affected by the lack of comprehensive data and standardized longitudinal follow-up, a consequence of patients’ remote residence and variable therapy compliance, which precludes a definitive assessment of disease progression or treatment efficacy. Additionally, the limited availability of maternal MLPA testing and Senger sequencing restricted our ability to perform thorough segregation analysis, thereby obscuring inheritance patterns.

## 5. Conclusions

Modern treatment approaches lead to expanded therapeutic options for children with DMD. However, delayed diagnosis significantly limits patients’ ability to maintain motor function, directly impacting their quality of life. With an average diagnostic age of 6–7.5 years, patients often lose the opportunity to benefit from Translarna, exon-skipping therapies, glucocorticoids, cardioprotective agents, and other symptomatic treatments within just a few years of diagnosis. This is precisely why it is essential to inform both the medical community (pediatricians, neurologists, gastroenterologists, geneticists, infectious disease specialists, and cardiologists) and the general public about the availability of DMD therapies.

Due to the presence of a pilot program of DMD/BMD screening of boys aged 12–14 months in St. Petersburg, we do not exclude the possibility of including this pathology in mass or selective screening throughout the Russian Federation. If it is not possible, we strongly recommend optimizing the diagnostic algorithm to include mandatory creatine kinase (CK) testing for all patients with even minimal suspicion of myopathy or elevated transaminase levels.

While primary diagnosis and treatment of DMD are adequately managed at the regional level, the long-term patient monitoring system requires optimization. This includes establishing a dedicated orphan disease center or neuromuscular specialty clinic, along with specialized training for local providers in standardized assessment protocols.

While current treatments significantly improve the quality of life for many DMD/BMD patients, they do not help everyone. A particular challenge remains with rare DMD gene variants that currently lack approved therapies. Nevertheless, the medical community and affected families remain optimistic that ongoing international research initiatives will yield targeted therapies for these patient subgroups soon.

## Figures and Tables

**Table 1:** Patients with DMD in RNOA.

Number of Patient	Gender	Ethnicity	Genotype (Based on NM_004006.3)	Age of Onset (Years)	Age of Diagnosis (Years)	Age of Last Exam (Years)	CK * (U/L)	Corticosteroid Therapy	Family History
1.1	m *	Ossetian	c.10141C > T (p.R3381*)	2	11	16	4133	withdrawal	mother’s brother had DMD, died at 18 y, male sibling died at 17 y (DMD?)
2.1	m	Ossetian	dup ex 46–64	2	7	15	3565	from the age of 7, prednisone 15 mg × 3/d, irregular	sporadic
3.1	m	Ossetian	del ex 7 and dup ex 5–6	5	9	14	16,283	from the age of 10, deflazacort, 15 mg/d	sporadic
4.1	m	Ossetian	del ex 43	2	5	13	51,554	from the age of 8, prednisone 15 mg × 3/d, irregular	mother’s brother had DMD, died at 15 y
5.1	m	Ossetian	del ex 46–47	2	4	12	26,013	from the age of 4, deflazacort, 12 mg/d	sporadic
6.1	m	Georgian/Ossetian	del ex 48–52	1	8	12	4913	withdrawal	sporadic
7.1	f *	Russian	del ex 3–11	4	10	12	26,908	n/d	sporadic
8.1	m	Ossetian	del ex 52	2	8	12	33,191	from the age of 9, prednisone 20 mg/d × 6 mo, then Eteplirsen	sporadic
9.1	m	Ossetian	del ex 45	6	7	11	14,013	from the age of 8, prednisone 15 mg × 3/d from the age of 10, deflazacort, 12 mg/d	sporadic
10.1	m	Russian	dup ex 17–19	3	8	11	37,077	from the age of 9, prednisone 15 mg × 3/d	sporadic
11.1	m	Ossetian	dup ex 2–9 and dup ex 54–56	2	4	11	9876	withdrawal	affected mother’s brother
12.1	m	Ossetian	dup ex 3–11	1.5	7	11	7369	from the age of 8, prednisone 12.5 mg × 3/d	n/d
13.1	m	Ossetian	del ex 8–17	2	5	10	13,565	from the age of 6, deflazacort, 12 mg/d	affected brother
13.2	m	Ossetian	del ex 8–17 47,XXY	n/d *	n/d	n/d	332	no clinical symptoms (47,XXY)	affected brother
14.1	m	Ossetian	c.9337C > T (p.R3113*)	1.5	9	9	7000	from the age of 9, prednisone 12.5 mg × 3/d	sporadic
15.1	m	Ossetian	c.6292 > T (p.R2098*)	3	9	9	13,710	from the age of 6, deflazacort, 16 mg/d	affected brother
15.2	m	Ossetian	c.6292 > T (p.R2098*)	2	7	9	13,100	from the age of 8, deflazacort, 12 mg/d	affected brother
16.1	m	Ossetian	c.2896C > T (p.Q966*)	1.5	4	8	18,002	from the age of 4, prednisone 12.5 mg × 3/d with Translarna	mother’s male cousin has a gait disturbance
17.1	m	Russian	del ex 46–50	1	2	7	14,565	from the age of 5, deflazacort, 12 mg/d	sporadic
18.1	m	Ossetian	c.7693C > T (p.Q2565*)	6	7	7	9094	from the age of 8, prednisone 16 mg/d	2 affected brothers
18.2	m	Ossetian	c.7693C > T (p.Q2565*)	4	8	6	17,940	from the age of 9, deflazacort, 21 mg/d	2 affected brothers
18.3	m	Ossetian	c.7693C > T (p.Q2565*)	2	3	3	17,332	from the age of 4, deflazacort, 16 mg/d	2 affected brothers
19.1	m	Ossetian	del ex 46–52	1.5	2	5	15,595	from the age of 3, deflazacort, 12 mg/d	sporadic
20.1	m	Ossetian	del ex 50–52	n/d	12	12	1486	n/d	n/d
21.1	m	Ossetian	del ex 45	n/d	5	5	25,013	n/d	n/d
22.1	m	Ossetian	del ex 32–43	n/d	6	6	14,444	n/d	n/d
23.1	m	Ossetian	del ex 45–50	6	8	8	1091	withdrawal	mother’s brother had DMD, died at 18 y
24.1	m	Ossetian	del ex 45	1.5	5	5	2565	withdrawal	sporadic

m—male; f—female; CK—creatine phosphokinase; n/d—no data; del—deletion; dup—duplication.

**Table 2:** Patient genotypes and types of target therapies.

Number of Patient	Genotype	Targeted Therapy	Age at Initiation of Therapy	Test Results Before Therapy	Age of Exam on Therapy	Test Results on Therapy
5.1	del 46–47	Casimersen	13 y	6 min WT *—417 m NSAA *-33/34	n/d	n/d
6.1	del 48–52	Viltolarsen	9 y 6 mo	n/d *	9 y 9 mo	NSAA—8/34; 6 min WT—178 m
8.1	del 52	Eteplirsen	11 y	n/d	12 y	n/d
15.1	c.6292 >T (p.R2098*)	Translarna	5 y	n/d	7 y 5 mo	6 min WT—450 m NSAA—27/34
15.2	c.6292 >T (p.R2098*)	Translarna	7 y	n/d	9 y	6 min WT—330 m NSAA—15/34
16.1	c.2896C > T (p.Q966*)	Translarna	4 y 6 mo	6 min WT—372 m NSAA—26/34	8 y 5 mo	6 min WT—454 m NSAA—26/34
17.1	del 46–50	Delandystrogen moxeparvovec-rokl	8 y	6 min WT—380 m	n/d	n/d
18.1	c.7693C > T (p.Q2565*)	Translarna	7 y	3 min WT—60 m NSAA—14/34	n/d	n/d
18.2	c.7693C > T (p.Q2565*)	Translarna	6 y	3 min WT—173 m NSAA—20/34	n/d	n/d
18.3	c.7693C > T (p.Q2565*)	Translarna	3 y 4 mo	3 min WT—75 m NSAA—19/34	n/d	n/d
19.1	del 46–52	Delandystrogen moxeparvovec-rokl	5 y 6 mo	6 min WT—392 m NSAA—17/34	5 y 10 mo	6 min WT—422 m NSAA—21/34

WT—3 or 6 min walk test; NSAA—North Star Ambulatory Assessment; n/d—no data.

## Data Availability

The datasets used and/or analyzed during the current study are available from the corresponding author upon reasonable request.

## Abbreviations

The following abbreviations are used in this manuscript:

DMD	Duchenne muscular dystrophy
RNOA	Republic of North Ossetia-Alania
RCCH	Republican Children’s Clinical Hospital
RF	Russian Federation
BMD	Becker muscular dystrophy
ALT	Alanine aminotransferase
AST	Aspartate aminotransferase
CK	Creatine phosphokinase
CS	Corticosteroid
LDH	Lactate dehydrogenase
MWT	Minute walk test
NSAA	North Star Ambulatory Assessment
NGS	Next generation sequencing
MLPA	Multiplex ligation-dependent probe amplification
RCMG	Research Centre for Medical Genetics
NMD	Nonsense-mediated decay
